# Assessment of knowledge and awareness of stroke among Arabic speaking adults: unveiling the current landscape in seven countries through the first international representative study

**DOI:** 10.3389/fneur.2024.1492756

**Published:** 2024-11-22

**Authors:** Diana Malaeb, Sara Mansour, Muna Barakat, Sarah Cherri, Zelal J. Kharaba, Feras Jirjees, Reem Al Zayer, Eyman M. Eltayib, Zeinab Khidhair, Hala AlObaidi, Sami El Khatib, Ruth Alex, Vineetha Menon, Basile Hosseini, Jinane Noureldine, Yassen Alfoteih, Souheil Hallit, Hassan Hosseini

**Affiliations:** ^1^College of Pharmacy, Gulf Medical University, Ajman, United Arab Emirates; ^2^Lebanese International University, School of Pharmacy, Beirut, Lebanon; ^3^Faculty of Pharmacy, Applied Science Private University, Amman, Jordan; ^4^College of Pharmacy, University of Sharjah, Sharjah, United Arab Emirates; ^5^Clinical Pharmacy Practice, Mohammed Al-Muna College for Medical Sciences, Dammam, Saudi Arabia; ^6^College of Pharmacy, Jouf University, Sakaka, Saudi Arabia; ^7^College of Science, University of Baghdad, Baghdad, Iraq; ^8^School of Pharmacy, Queens University Belfast, Belfast, United Kingdom; ^9^Department of Biomedical Sciences, Lebanese International University, Bekaa, Lebanon; ^10^Center for Applied Mathematics and Bioinformatics (CAMB), Gulf University for Science and Technology, Mubarak Al-Abdullah, Kuwait; ^11^Hospices Civils de Lyon, Lyon, France; ^12^Rammal Hassan Rammal Research Laboratory, PhyToxE Research Group, Nabatieh, Lebanon; ^13^Faculty of Sciences, Lebanese University, Nabatieh, Lebanon; ^14^College of Dental Surgery, City University Ajman, Ajman, United Arab Emirates; ^15^College of General Education, City University Ajman, Ajman, United Arab Emirates; ^16^School of Medicine and Medical Sciences, Holy Spirit University of Kaslik, Jounieh, Lebanon; ^17^Applied Science Research Center, Applied Science Private University, Amman, Jordan; ^18^UPEC-University Paris-Est, Creteil, France; ^19^RAMSAY SANTÉ, HPPE, Champigny sur Marne, France

**Keywords:** stroke, knowledge, awareness, community, risk factors, MENA region stroke, MENA region, symptom identification

## Abstract

**Introduction:**

While several studies have examined stroke public knowledge and awareness in individual countries within the Middle East and North Africa (MENA) region, none have provided a comprehensive cross-country assessment.

**Purpose:**

To assess public stroke knowledge and awareness among Arabic-speaking adults in seven MENA countries and identify associated factors.

**Materials and methods:**

An online cross-sectional survey was self-administered by the public population in Iraq, Lebanon, Sudan, Jordan, United Arab Emirates, Syria, and Saudi Arabia (April 2021–2023). Associations of stroke risk factors, early symptoms, and consequences with socio-demographics and medical history were analyzed using logistic regression models.

**Results:**

Of 4,090 participants (58.3% females), 42.9% identified four out of five correct answers related to general stroke knowledge. Only 25.2% identified all stroke risk factors, 24.7% recognized all symptoms, and 37.5% knew all possible consequences. Results show consistent pattern of high identification for at least one risk factor and consequences across all countries (96.3 to 99.8% and 86.2 to 100%, respectively), with varying levels of early symptom identification (56.8 to 97.9%). Females were more likely to identify a stroke risk factor, symptom, and consequence compared to males (OR = 2.525, 2.474, and 2.302, respectively, *p* < 0.001). Employed, urban residents, and those with higher education demonstrated better stroke awareness.

**Conclusion:**

The sample showed variable levels of stroke knowledge among the public, underscoring the pressing need for targeted community initiatives, media campaigns, and educational interventions. These efforts are paramount for improving awareness, early detection, and timely response, especially in countries with lower levels of community stroke awareness.

## 1 Introduction

Stroke is a significant global health concern and contributes to mortality, morbidity, and disability worldwide (1). Stroke ranks fifth among all causes of death with the highest likelihood occurring within 1 to 5 years after stroke in individuals aged 75 years and older (2). According to the American Health Association 2024, around 795,000 people experience a new or recurrent stroke, with about 610,000 of these as first attacks and 185, 000 as recurrent attacks in the United States (2).

The burden of stroke has decreased in the Middle East and North Africa (MENA) region over the past three decades with large intercountry differences. In 2019, the prevalence and mortality rates of stroke in the MENA region showed a slight decrease of 0.5% in prevalence and a significant 27.8% decline in mortality since 1990 (3).

Despite these improvements, a lack of knowledge and awareness about modifiable and non-modifiable stroke risk factors continues to contribute to the burden of stroke (4). Early warning symptoms of stroke are important for timely management and better treatment outcomes. Rapid thrombolysis therapy during the first 4–6 h from the onset of stroke symptoms lowers the incidence of disabilities and enhances clinical outcomes in patients with ischemic stroke (5). Studies have shown that failure to identify stroke symptoms can delay timely treatment, leading to physical complications, mental disabilities, and increased mortality (4, 6).

There is a consistent trend across studies conducted in countries within the MENA region, including Iraq, Lebanon, Sudan, Jordan, and the United Arab Emirates (UAE), where females demonstrated a higher proficiency in identifying at least one risk factor of stroke (7–11). However, in Saudi Arabia, males showed a significantly higher ability to identify at least one risk factor of stroke compared to females (8). Additionally, in the UAE, Saudi Arabia, Lebanon, Syria, and Jordan, individuals with a university degree, demonstrated greater ability to identify either early stroke symptoms or the consequences of stroke (7–11). In the UAE, individuals with diabetes mellitus were more likely to recognize at least one consequence of stroke compared to patients without diabetes mellitus (7). In Saudi Arabia, individuals with a history of hypertension, dyslipidemia, and obesity were able to identify at least one early stroke symptom (8). However, in Iraq and Jordan, diabetic patients exhibited significantly lower odds of recognizing stroke symptoms compared to non-diabetic patients (9, 11).

Community-based educational initiatives are paramount for stroke prevention by raising patient awareness and knowledge. Adequate knowledge improves quality of life, lowers risk of recurrent strokes, decreases hospitalizations, and reduces healthcare burden and costs (12, 13). Furthermore, increasing public knowledge and awareness of early warning symptoms ensures timely management of stroke, and improves outcomes (7, 8).

While there have been several studies on stroke knowledge and awareness in individual countries within the MENA region, none has provided a comprehensive assessment across countries. Therefore, this study aims to address this gap by assessing public knowledge and awareness of stroke and identifying factors associated with stroke awareness across seven countries in the MENA region. Our study will provide valuable insights to develop targeted educational programs and interventions for stroke prevention and management strategies in the MENA region.

## 2 Materials and methods

### 2.1 Study design and participants

This cross-sectional study was conducted using an anonymous online survey in Iraq, Lebanon, Sudan, Jordan, the United Arab Emirates (UAE), Syria, and Saudi Arabia. The snowball sampling method was used from April 2021 to 2023. The data collection sheet was developed on Google form and an electronic link was distributed to the public population in each country through digital platforms such as WhatsApp, LinkedIn, and Facebook. Participation was voluntary. Participants over 18 years of age were eligible; individuals with a history of stroke were excluded.

Participants’ anonymity was guaranteed during the data collection process. At the beginning of the survey, participants were provided with a written informed consent form titled “Your participation in completing this survey is highly appreciated.” Participants provided their electronic consent if they wished to continue with the survey. If not, they selected “disagree to participate” and did not continue the survey. Potential participants who completed the survey were deemed to have provided informed consent to participate in the study.

### 2.2 Study tool

The survey was written in Arabic, the native language of the included countries, and designed in simple Arabic Language. Pilot was done before study initiation in the involved countries to standardize the data collection form and to ensure it is validity. The data collected for the pilot were not used in the study. The estimated time to complete the questionnaire is 15 min. This investigation was conducted based on previous literature (14, 15). Participants completed without assistance from the investigators to avoid possible influence on answering questions.

The study tool consists of two main parts: the first part of the questionnaire covered sociodemographic and socioeconomic factors, including age, smoking status, marital status, employment status, household income, place of residence, educational level, and medical history (16). The second section assessed common knowledge about stroke. Respondents answered the following statements: Stroke (1) affects the brain, (2) is common in older adults, (3) is contagious, (4) is hereditary, and (5) is preventable. This section also assesses awareness of stroke risk factors, including hypertension, smoking, alcohol consumption, dyslipidemia, diabetes mellitus, physical inactivity, heart disease, obesity, age, and psychosocial stress. Additionally, awareness of early warning signs was examined: (1) Sudden numbness or weakness in the face/arms/legs, especially on one side of the body; (2) Sudden confusion or difficulty speaking/understanding speech; (3) Sudden numbness/weakness in one or both eyes Sudden visual impairment; (4) Sudden difficulty walking, dizziness, or loss of balance or coordination; (5) Sudden severe headache of unknown origin. In line with previous research by Han et al. (15).

Participants received one point for each correct answer to the above statements, but cutoffs to determine acceptable levels of knowledge were lacking. Therefore, our study calculated the total knowledge score by summing up the total number of correct answers.

### 2.3 Statistical analysis

All study data was extracted from the Google form as Excel spreadsheet and imported to the Statistical Package for Social Sciences version (SPSS) 25.0 for analysis. Categorical variables were presented as frequencies (*n*) and percentages (%) and continuous variables as means with standard deviation (SD). Bivariate associations between risk factors, early symptoms, and consequences of stroke with socio-demographics and medical history were analyzed using Chi-square test (or Fisher’s exact test if cell count was less than five). Binary logistic regression was performed to determine factors associated with the ability to spontaneously identify at least one or more stroke risk factors, one or more warning signs, one or more consequences, and seeking an emergency room as soon as stroke develops. Variables with a *p* < 0.2 in the bivariate analysis were included in the logistic regression models. Results were presented as odds ratios (OR) and 95% confidence interval. Statistical tests were reported statistically significant at *p* < 0.05.

## 3 Results

### 3.1 Sample description

Of the total 4,090 participants enrolled in the study, 2,380 (58.3%) were females, 1789 (43.8%) were under 30 years, almost half were single (44.4%), and most of them were residing in urban areas and had university level of education (74.9 and 73.1% respectively). The most common concomitant disease was hypertension (22.6%), followed by dyslipidemia (20.8%) and peptic ulcer (20.0%). The majority of the participants have heard of stroke as a disease (91.3%) and 70.5% knew someone with a stroke. Sociodemographic factors and familiarity with stroke are displayed in Table 1.

**Table 1:** Participants’ sociodemographic characteristics, past medical history, and familiarity with stroke.

Variables (*N* = 4,090)		Frequency (%)
Sociodemographic characteristics		
Gender	Male	1700 (41.6)
	Female	2,380 (58.3)
Age (years)	<30	1789 (43.8)
	30–49	1,678 (41.1)
	>50	620 (15.2)
Residence area	Urban	2,745 (74.9)
	Rural	918 (25.1)
Marital status	Single	1813 (44.4)
	Married	1797 (44.0)
	Divorced	265 (6.5)
	Widowed	211 (5.2)
Educational level	School level	1,069 (26.9)
	University level	2,908 (73.1)
Employment status	Unemployed	1779 (43.6)
	Employed	2,303 (56.4)
Income level	Low	1,665 (42.7)
	Medium	1,442 (37.0)
	High	793 (20.3)
History of smoking (≥1 year)	Yes	1726 (42.4)
Past medical history	Hypertension	919 (22.6)
	Diabetes Mellitus	429 (10.6)
	Dyslipidemia	844 (20.8)
	Arrhythmia	759 (18.7)
	Kidney disease	476 (11.8)
	Peptic ulcer	810 (20.0)
	Depression	748 (18.5)
	Obesity	665 (16.6)
Familiarity with stroke	Ever heard of stroke	3,233 (91.3)
	History of stroke in the family	1,150 (28.2)
	Personally know someone with stroke	2,881 (70.5)

### 3.2 Stroke knowledge, risk factors, early symptoms, and consequences

**Figure 1:**
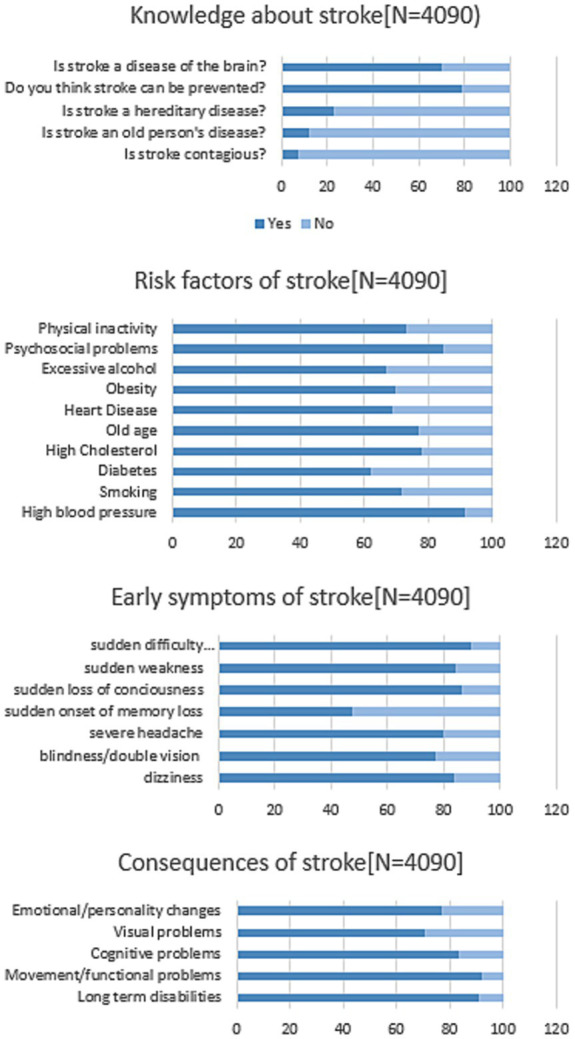
Proportion (%) of responses regarding stroke knowledge, risk factors, and early symptoms.

The sample showed a variable level of knowledge about stroke (Figure 1; Table 2). More than half of the participants were aware that stroke is a disease of the brain and that it can be prevented (70.1 and 78.7%, respectively). About 42.9% of the participants could identify four out of five correct answers related to general stroke knowledge. Furthermore, 91.6% believed that hypertension was the most common risk factor, followed by psychosocial stress (84.7%) and high cholesterol (78.1%; Figure 1). The most commonly identified symptoms by participants were “Sudden difficulty in speaking or understanding speech” and “sudden loss of consciousness,” accounting for 89.7 and 86.7%, respectively. Only 25.2% identified all stroke risk factors, 24.7% recognized all stroke early symptoms, and 37.5% knew all possible consequences of stroke (Table 2).

**Table 2:** Number of stroke risk factors, early symptoms, and consequences that were identified by the participants.

Variables		Frequency (%)	Cumulative, Frequency (%)
Number of correct answers regarding stroke in the general knowledge	Zero	19 (0.5)	19 (0.5)
	One	127 (3.1)	146 (3.6)
	Two	370 (9.1)	516 (12.7)
	Three	1,062 (26.1)	1,578 (38.7)
	Four	1748 (42.9)	3,326 (81.6)
	Five	748 (18.4)	4,074 (100)
Number of identified risk factors of stroke	Zero	57 (1.7)	57 (1.7)
	One	56 (1.6)	113 (3.3)
	Two	64 (1.9)	177 (5.1)
	Three	336 (9.8)	513 (14.9)
	Four	301 (8.7)	814 (23.6)
	Five	455 (13.2)	1,269 (36.9)
	Six	360 (10.5)	1,629 (47.3)
	Seven	278 (8.1)	1907 (55.4)
	Eight	314 (9.1)	2,221 (64.5)
	Nine	353 (10.3)	2,574 (74.8)
	Ten	869 (25.2)	3,443 (100)
Number of identified early symptoms of stroke	Zero	182 (4.5)	182 (4.5)
	One	60 (1.5)	242 (6.0)
	Two	201 (5.0)	443 (11.0)
	Three	388 (9.7)	831 (20.7)
	Four	581 (14.5)	1,412 (35.1)
	Five	862 (21.4)	2,274 (56.6)
	Six	752 (18.7)	3,026 (75.3)
	Seven	993 (24.7)	4,019 (100)
Number of identified consequences of stroke	Zero	156 (3.8)	156 (3.8)
	One	97 (2.4)	253 (6.2)
	Two	418 (10.3)	671 (16.6)
	Three	918 (22.6)	1,589 (39.2)
	Four	946 (23.3)	2,535 (62.5)
	Five	1,519 (37.5)	4,054 (100)

### 3.3 Cross-country comparison

The study shows a consistent pattern of high identification of at least one stroke risk factor across all seven studied countries, ranging from 96.3 to 99.8% (Table 3).

**Table 3:** Identification of stroke risk factors, early symptoms, and consequences by the participants across the seven countries.

	Risk factor(s) identified (≥1)		Early symptom(s) identified (≥1)		Consequence(s) identified (≥1)	
	Yes	No	Yes	No	Yes	No
Lebanon (*N* = 551)	533 (97.8)	12 (2.2)	520 (95.9)	22 (4.1)	520 (95.9)	22 (4.1)
Syria (*N* = 1,013)	996 (98.3)	17 (1.7)	992 (97.9)	21 (2.1)	999 (98.6)	14 (1.4)
UAE (*N* = 545)	544 (99.8)	1 (0.2)	490 (89.9)	55 (10.1)	470 (86.2)	75 (13.8)
Jordan (*N* = 573)	562 (98.1)	11 (1.9)	547 (95.5)	26 (4.5)	562 (98.1)	11 (1.9)
Iraq (*N* = 609)	599 (98.4)	10 (1.6)	582 (95.6)	27 (4.4)	583 (95.7)	26 (4.3)
Sudan (*N* = 410)	395 (96.3)	15 (3.7)	379 (92.4)	31 (7.6)	390 (95.1)	20 (4.9)
Saudi Arabia (*N* = 389)	387 (99.5)	2 (0.5)	221 (56.8)	168 (43.2)	389 (100)	0

Regarding the identification of at least one early stroke symptom, this study reveals varying patterns across countries. Lebanon, Syria, Jordan, Iraq, and Sudan demonstrate relatively high levels of early symptom identification, ranging from 92.4 to 97.9%. However, the UAE shows a lower level of symptom identification at 89.9%, and Saudi Arabia reports the lowest at 56.8%. Syria, Jordan, and Saudi Arabia show notably high levels of stroke consequence recognition, with percentages ranging from 98.1 to 100%. Lebanon, Iraq, and Sudan also exhibit strong identification exceeding 95%. However, the UAE presents a lower rate at 86.2%.

### 3.4 Bivariate analysis

A significantly higher proportion of females versus males correctly identified risk factors (58.6% vs. 41.4% *p* = 0.007), symptoms (95.2% vs. 86.4% *p* < 0.001), and consequences of stroke (96.8% vs. 95.3% *p* = 0.016; Table 4). A significantly higher proportion of subjects aged below 30 years versus other age groups (*p* < 0.001) and who lived in urban versus rural areas (92.5% vs. 88.6%, *p* < 0.001) correctly identified at least one early symptom of stroke. Furthermore, single and widowed participants and those with higher educational levels exhibited highest percentage of correct responses about early stroke symptoms (*p* < 0.001). On the other hand, unemployed, those with a high-income level, and with no history of smoking recognized at least one warning symptom of stroke (*p* < 0.001). Moreover, participants with no history of hypertension (*p* = 0.007), diabetes mellitus (*p* < 0.001), arrhythmia (*p* = 0.001), peptic ulcer (92.1%; *p* = 0.002), kidney disease (*p* < 0.001), peptic ulcer (*p* = 0.002) and depression (*p* < 0.001) showed the highest percentage of correctly answering at least one question about stroke early symptoms.

**Table 4:** Association of risk factors, early symptoms, and consequences of stroke with the sociodemographic characteristics and past medical history.

Variables (*N* = 4,090)		Risk factor(s) identified (≥1)			Early symptom(s) identified (≥1)			Consequence(s) identified (≥1)		
		Yes (*n* = 4,016), (98.6%)	No (*n* = 57), (1.4%)	*p*-value	Yes (*n* = 3,731), (91.5%)	No (*n* = 348), (8.5%)	*p*-value	Yes (*n* = 3,898), (96.2%)	No (*n* = 156), (3.8%)	*p*-value
Sociodemographic characteristics										
Gender	Male	1,660 (41.4)	34 (59.6)	**0.007**	1,467 (86.4)	230 (13.6)	**<0.001**	1,611 (95.3)	80 (4.7)	**0.016**
	Female	2,347 (58.6)	23 (40.4)		2,259 (95.2)	114 (4.8)		2,278 (96.8)	76 (3.2)	
Age (years)	<30	1754 (98.7)	24 (1.3)	0.983	1,685 (94.6)	97 (5.4)	**<0.001**	1,683 (95.0)	88 (5.0)	**<0.001**
	30–49	1,651 (98.6)	24 (1.4)		1,486 (88.6)	191 (11.4)		1,609 (96.4)	60 (3.6)	
	> 50	609 (98.5)	9 (1.5)		558 (90.3)	60 (9.7)		604 (98.7)	8 (1.3)	
Residence area	Urban	2,691 (98.5)	42 (1.5)	0.878	2,535 (92.5)	207 (7.5)	**<0.001**	2,665 (97.7)	63 (2.3)	**<0.001**
	Rural	899 (98.4)	15 (1.6)		807 (88.6)	104 (11.4)		854 (94.7)	48 (5.3)	
Marital status	Single	1772 (98.3)	31 (1.7)	0.191	1716 (95.0)	91 (5.0)	**<0.001**	1718 (95.5)	81 (4.5)	**0.008**
	Married	1769 (98.7)	23 (1.3)		1,578 (88.0)	215 (12.0)		1707 (96.1)	70 (3.9)	
	Divorced	262 (98.9)	3 (1.1)		232 (87.5)	33 (12.5)		262 (98.9)	3 (1.1)	
	Widowed	210 (100)	0 (0)		202 (95.7)	9 (4.3)		208 (99.0)	2 (1.0)	
Educational level	School	1,049 (98.5)	16 (1.5)	0.880	863 (81.0)	202 (19.0)	**<0.001**	1,016 (95.8)	45 (4.2)	0.456
	University	2,857 (98.6)	41 (1.4)		2,759 (95.1)	143 (4.9)		2,774 (96.3)	107 (3.7)	
Employment status	Unemployed	1736 (98.3)	30 (1.7)	0.179	1,653 (93.4)	116 (6.6)	**<0.001**	1,689 (96.0)	71 (4.0)	0.622
	Employed	2,273 (98.8)	27 (1.2)		2071 (89.9)	232 (10.1)		2,202 (96.3)	85 (3.7)	
Income level	Low	1,643 (98.9)	18 (1.1)	0.519	1,481 (89.2)	67 (4.6)	**<0.001**	1,595 (96.5)	58 (3.5)	0.603
	Medium	1,415 (98.5)	22 (1.5)		1,329 (92.2)	56 (4.4)		1,384 (96.8)	46 (3.2)	
	High	777 (98.5)	12 (1.5)		749 (94.5)	44 (5.6)		760 (96.0)	32 (4.0)	
History of smoking (≥1 year)	No	2,304 (98.5)	36 (1.5)	0.273	2,202 (93.8)	146 (6.2)	**<0.001**	2,224 (95.5)	106 (4.5)	**0.008**
	Yes	1704 (98.8)	19 (1.1)		1,521 (88.3)	201 (11.7)		1,670 (97.1)	50 (2.9)	
Past medical history										
Hypertension	No	3,088 (98.6)	43 (1.4)	1.0	2,889 (92.1)	248 (7.9)	**0.007**	2,994 (95.8)	132 (4.2)	**0.031**
	Yes	905 (98.7)	12 (1.3)		820 (89.2)	99 (10.8)		892 (97.4)	24 (2.6)	
Diabetes Mellitus	No	3,561 (98.6)	51 (1.4)	0.515	3,334 (92.2)	284 (7.8)	**<0.001**	3,463 (96.0)	145 (4.0)	0.147
	Yes	423 (99.1)	4 (0.9)		367 (85.5)	62 (14.5)		416 (97.4)	11 (2.6)	
Dyslipidemia	No	3,156 (98.5)	48 (1.5)	0.179	2,943 (91.7)	266 (8.3)	0.240	3,064 (95.8)	136 (4.3)	**0.015**
	Yes	834 (99.2)	7 (0.8)		763 (90.4)	81 (9.6)		818 (97.6)	20 (2.4)	
Arrhythmia	No	3,239 (98.5)	49 (1.5)	0.164	3,033 (92.2)	258 (7.8)	**0.001**	3,139 (95.7)	142 (4.3)	**0.002**
	Yes	748 (99.2)	6 (0.8)		670 (88.3)	89 (11.7)		743 (98.2)	14 (1.8)	
Kidney disease	No	3,509 (98.6)	51 (1.4)	0.4	3,300 (92.5)	267 (7.5)	**<0.001**	3,410 (95.9)	146 (4.1)	**0.042**
	Yes	471 (99.2)	4 (0.8)		396 (83.2)	80 (16.8)		466 (97.9)	10 (2.1)	
Peptic ulcer	No	3,176 (98.5)	48 (1.5)	0.181	2,977 (92.1)	255 (7.9)	**0.002**	3,077 (95.5)	144 (4.5)	**<0.001**
	Yes	803 (99.1)	7 (0.9)		718 (88.6)	92 (11.4)		798 (98.6)	11 (1.4)	
Depression	No	3,250 (98.6)	43 (1.4)	0.564	3,076 (93.2)	225 (6.8)	**<0.001**	3,150 (95.8)	139 (4.2)	**0.015**
	Yes	735 (98.7)	10 (1.3)		624 (83.6)	122 (16.4)		729 (97.7)	17 (2.3)	
Obesity	No	3,292 (98.6)	48 (1.4)	0.476	3,049 (91.2)	296 (8.8)	0.110	3,207 (96.2)	128 (3.8)	0.435
	Yes	655 (98.9)	7 (1.1)		619 (93.1)	46 (6.9)		641 (96.8)	21 (3.2)	

Fisher’s exact test was used when the cell counts were less than 5. Numbers in bold indicate significant *p*-values.

A significantly higher proportion of subjects aged more than 50 years old (*p* < 0.001) and those who lived in urban areas vs. rural (97.7% vs. 94.7%; *p* < 0.001) correctly identified consequences emerging from stroke (Table 4). Moreover, widowed and divorced individuals (*p* = 0.008) and those with a history of smoking versus no history (97.1% vs. 95.5%; *p* = 0.008) demonstrated the highest percentage of accurately answering at least one question regarding stroke consequences. A significantly higher number of participants with a history of hypertension (*p* = 0.031), dyslipidemia (*p* = 0.015), arrhythmia (*p* = 0.002), kidney disease (*p* = 0.042), peptic ulcer disease (*p* < 0.001) and depression (*p* = 0.015) identified at least one correct answer about consequences (Table 4).

A significantly higher number of correct answers regarding taking patient to the hospital when there is a stroke symptom was associated with female gender (*p* < 0.001), younger age (*p* < 0.001), living in urban areas (80.3% vs. 63.9%, *p* < 0.001), single status (*p* < 0.001), university vs. school level of education (79.4% vs. 48.0%, *p* < 0.001), employed versus unemployed (72.9% vs. 69.3%, *p* = 0.010), high-income level versus lower income (*p* < 0.001), and those who had no history of smoking (*p* < 0.001; Table 5). In addition, those who had no history of hypertension, diabetes mellitus, dyslipidemia, arrhythmia, kidney disease, peptic ulcer, or depression also responded correctly regarding taking patients with symptoms to the hospital (*p* < 0.001 for all; Table 5).

**Table 5:** Association of taking a patient who is experiencing a stroke to the hospital with sociodemographic characteristics and past medical history.

Variables (*N* = 4,090)		Taking a patient who is experiencing a stroke to the hospital		
		Yes (*n* = 2,911), (71.2%)	No (*n* = 1,175), *n* (28.8%)	*p*-value
Sociodemographic characteristics				
Gender	Male	1,155 (67.9)	545 (32.1)	**<0.001**
	Female	1750 (73.6)	627 (26.4)	
Age (years)	<30	1,399 (78.3)	387 (21.7)	**<0.001**
	30–49	1,174 (70.0)	504 (30.0)	
	>50	337 (54.4)	283 (45.6)	
Residence area	Urban	2,204 (80.3)	540 (19.7)	**<0.001**
	Rural	585 (63.9)	331 (36.1)	
Marital status	Single	1,452 (80.2)	359 (19.8)	**<0.001**
	Married	1,241 (69.1)	555 (30.9)	
	Divorced	144 (54.3)	121 (45.7)	
	Widowed	72 (34.1)	139 (65.9)	
Educational level	School	513 (48.0)	555 (52.0)	**<0.001**
	University	2,306 (79.4)	600 (20.6)	
Employment status	Unemployed	1,230 (69.3)	546 (30.7)	**0.010**
	Employed	1,680 (72.9)	623 (27.1)	
Income level	Low	1,066 (64.0)	599 (36.0)	**<0.001**
	Medium	1,114 (77.3)	328 (22.7)	
	High	632 (80.2)	157 (19.8)	
History of smoking (≥1 year)	No	1742 (74.2)	607 (25.8)	**<0.001**
	Yes	1,160 (67.2)	566 (32.8)	
Past medical history				
Hypertension	No	2,383 (75.9)	758 (24.1)	**<0.001**
	Yes	510 (55.5)	409 (44.5)	
Diabetes Mellitus	No	2,641 (72.9)	981 (27.1)	**<0.001**
	Yes	243 (56.6)	186 (43.4)	
Dyslipidemia	No	2,402 (74.8)	811 (25.2)	**<0.001**
	Yes	486 (57.6)	358 (42.4)	
Arrhythmia	No	2,404 (73.0)	891 (27.0)	**<0.001**
	Yes	482 (63.5)	277 (36.5)	
Kidney disease	No	2,578 (72.2)	993 (27.8)	**<0.001**
	Yes	302 (63.4)	174 (36.6)	
Peptic ulcer	No	2,421 (74.8)	815 (25.2)	**<0.001**
	Yes	461 (56.9)	349 (43.1)	
Depression	No	2,447 (74.1)	857 (25.9)	**<0.001**
	Yes	437 (58.4)	311 (41.6)	
Obesity	No	2,417 (72.2)	932 (27.8)	0.220
	Yes	464 (69.8)	201 (30.2)	

Fisher’s exact test was used when the cell count was less than 5. Numbers in bold indicate significant *p*-values.

### 3.5 Multivariable analysis

Table 6 presents factors associated with the identification of at least one stroke risk factor, early symptom, and consequence. The multivariable analysis showed that females were more likely to identify a risk factor, symptom, and consequence of stroke compared to males (OR = 2.525, 2.474, and 2.302, respectively, *p* < 0.001; Table 6). Those employed were more likely to identify one stroke risk factor compared to those unemployed (OR = 1.957; 95%CI 1.102; 3.477, *p* = 0.022).

**Table 6:** Multivariable analysis.

Variables (*N* = 4,090)	*β* (SE)	OR (95% CI)	*p*-value
Risk factor(s) identified (≥1)			
Gender (female versus male*)	0.926 (0.289)	2.525 (1.434; 4.447)	**<0.001**
Employment status (employed versus unemployed*)	0.672 (0.293)	1.957 (1.102; 3.477)	0.022
Marital status			
Married vs. single*	0.153 (0.294)	1.165 (0.655; 2.072)	0.603
Divorced vs. single*	0.344 (0.615)	1.410 (0.422; 4.711)	0.577
Widowed vs. single*	17.247 (2736.7)	30,923,886 (0.0;0.0)	0.995
Early symptom(s) identified (≥1)			
Gender (female versus male*)	0.906 (0.153)	2.474 (1.833; 3.336)	**<0.001**
Age (Years)			
30–49 vs. <30	−0.444 (0.185)	0.641 (0.446; 0.922)	**0.017**
>50 vs. <30	−0.269 (0.240)	0.765 (0.477; 1.225)	0.264
Marital status			
Married vs. single*	−0.519 (0.188)	0.594 (0.411; 0.858)	**0.006**
Divorced vs. single*	0.111 (0.280)	1.118 (0.646; 1.935)	0.691
Widowed vs. single*	1.186 (0.445)	3.283 (1.371; 7.828)	**0.008**
Educational level (university versus school*)	1.319 (0.157)	3.738 (2.748; 5.086)	**0.001**
Income level			
Medium vs. low*	0.270 (0.149)	1.310 (0.978; 1.755)	0.070
High vs. low*	0.503 (0.216)	1.654 (1.082; 2.529)	**0.020**
History of smoking (≥1 year) (yes versus no*)	0.265 (0.155)	1.303 (0.961; 1.767)	0.089
Hypertension (yes versus no*)	0.491 (0.168)	1.633 (1.174; 2.272)	**0.004**
Diabetes Mellitus (yes versus no*)	−0.312 (0.182)	0.732 (0.512; 1.045)	0.086
Depression (yes versus no*)	−0.758 (0.151)	0.469 (0.349; 0.630)	**<0.001**
Obesity (yes versus no*)	0.557 (0.198)	1.745 (1.184; 2.574)	**0.005**
Consequence(s) identified (≥1)			
Gender (female versus male*)	0.834 (0.216)	2.302 (1.507; 3.517)	**<0.001**
Age			
30–49 vs. <30*	0.571 (0.218)	1.770 (1.154; 2.713)	**0.009**
≥50 vs. <30*	0.928 (0.386)	2.530 (1.186; 5.395)	**0.016**
Residence area (rural versus urban*)	−0.907 (0.200)	0.404 (0.273; 0.597)	**<0.001**
History of smoking (≥1 year) (yes versus no*)	0.916 (0.239)	2.500 (1.564; 3.997)	**<0.001**
Peptic ulcer disease (yes versus no*)	0.680 (0.330)	1.974 (1.034; 3.768)	**0.039**
Taking a patient to a hospital			
Age			
30–49 vs. <30*	0.093 (0.130)	1.098 (0.850; 1.417)	0.475
≥50 vs. <30*	−0.425 (0.166)	0.654 (0.472; 0.905)	**0.010**
Residence area (rural versus urban*)	−0.761 (0.102)	0.467 (0.383; 0.571)	**<0.001**
Marital status			
Married vs. single*	−0.270 (0.131)	0.763 (0.590; 0.987)	**0.040**
Divorced vs. single*	−0.498 (0.193)	0.607 (0.416; 0.886)	**0.010**
Widowed vs. single*	−1.427 (0.233)	0.240 (0.152; 0.379)	**<0.001**
Educational level (university versus school*)	1.269 (0.107)	3.557 (2.882; 4.390)	**<0.001**
Hypertension (yes versus no*)	−0.303 (0.116)	0.739 (0.588; 0.928)	**0.009**
Kidney disease (yes versus no*)	0.275 (0.140)	1.316 (1.000; 1.732)	**0.050**
Peptic ulcer disease (yes versus no*)	−0.312 (0.113)	0.732 (0.586; 0.913)	**0.006**
Depression (yes versus no*)	−0.594 (0.118)	0.552 (0.438; 0.696)	**<0.001**

β, Beta; SE, standard error; OR; adjusted ratio; CI, confidence interval. Logistic regression taking identification of stroke risk factors, stroke early symptoms, stroke consequences, taking a patient who is experiencing stroke to the hospital as the dependent variables and sociodemographic factors and medical history as independent variables.

Regarding the identification of at least one early stroke symptom, individuals aged 30–49 years versus <30 years (OR = 0.641; 95%CI 0.446; 0.922, *p* = 0.017), married versus single (OR = 0.594; 95%CI 0.411; 0.858, *p* = 0.006) and those with a history of depression versus no history (OR = 0.469; 95%CI 0.349; 0.630, *p* < 0.001) had significantly lower odds of identifying at least one correct early stroke symptom. Whereas, individuals with higher educational attainment (OR = 3.738, 95%CI 2.748; 5.086, *p* = 0.001), widowed compared to single (OR = 3.283; 95%CI 1.371; 7.828, *p* = 0.008), those with high income versus lower income levels (OR = 1.654; 95%CI 1.082; 2.529, *p* = 0.020) and those with history of hypertension and obesity (OR = 1.633, *p* = 0.004 and OR = 1.745, *p* = 0.005, respectively; Table 6) had significantly higher odds identifying at least one stroke symptom.

Concerning the identification of at least one consequence of stroke, older individuals with 30–49 years old and ≥ 50 years compared with <30 years (OR = 1.770; *p* = 0.009 and OR = 2.530; *p* = 0.016, respectively), those with a history of smoking (OR = 2.500; *p* < 0.001), and peptic ulcer disease (OR = 1.974; *p* = 0.039) had significantly higher odds of identifying a stroke consequence. Individuals living in rural areas had significantly lower odds of identifying a correct stroke consequence compared to those living in urban areas (OR = 0.404; 95% CI 0.273; 0.597, *p* < 0.001; Table 6).

For the response to stroke symptoms, individuals with a university degree compared to those with lower education (OR = 3.557; 95% CI 2.882; 4.390, *p* < 0.001; Table 6) were more likely to take a patient experiencing stroke symptoms to a hospital. However, individuals of older age (OR = 0.654), living in rural areas (OR = 0.467), those divorced (OR = 0.607), married (OR = 0.763) or widowed (OR = 0.240) compared to single, and those with chronic disease [i.e., history of hypertension (OR = 0.739), peptic ulcer (OR = 0.732) or depression (OR = 0.552)] had significantly lower odds of responding by taking a patient experiencing stroke symptoms to the hospital.

## 4 Discussion

### 4.1 Main findings

This multi-country study assessed public awareness of stroke, focusing on knowledge and identification of stroke risk factors, early warning symptoms, and consequences. Our results showed a variable level of knowledge about stroke and females, employed individuals, urban residents, and those with higher educational attainment demonstrated better knowledge of stroke-related information. Also, 25.2% of individuals identified all stroke risk factors, 24.7% recognized all stroke early symptoms, and 37.5% knew all possible consequences of stroke. Despite this, findings revealed high public awareness of at least one stroke risk factor in all seven countries. However, the identification of at least one early stroke symptom varied: Lebanon, Syria, Jordan, Iraq, and Sudan showed relatively high levels while the UAE and Saudi Arabia had lower levels. Syria, Jordan, and Saudi Arabia reported high recognition of stroke consequences, along with Lebanon, and Iraq, while the UAE had lower rates. Overall, these findings highlight the pressing need for targeted educational interventions and healthcare initiatives to enhance public awareness of stroke, particularly in regions with lower community awareness. Improved knowledge can empower individuals to recognize warning symptoms promptly, seek timely management, and ultimately reduce morbidity and mortality associated with strokes. Addressing these awareness gaps can significantly impact public health outcomes by mitigating stroke burden.

### 4.2 Stroke general knowledge

The study demonstrated that the majority of the participants were aware of stroke, closely aligning with Dar et al., who reported that 80.5% of patients were informed about the condition (17). This contrasts with several studies that reported lower awareness levels (18–22). The relatively high awareness in our study likely stems from the urban and educated nature of the sample, which promotes greater information exposure and facilitates effective knowledge sharing through close interpersonal and family ties, highlighting the influence of demographic and social factors on public health education aimed at improving stroke awareness within communities. Among those surveyed, 42.9% correctly identified four out of five stroke-related general knowledge, with 18.4% answering all questions, though 0.5% could not provide any correct answers. Notably, 70.1% recognized stroke as a brain disease, and 78.7% acknowledged its preventability. However, several misconceptions about stroke persisted; 23.2% mistakenly considered it hereditary, 12.2% thought it affected only the elderly, and 7.3% incorrectly believed it to be contagious. Similarly, Dar et al. found that 76.0% understood stroke as a brain disease, 85.4% believed in its preventability, but 43.8% considered it hereditary, 32.6% thought it primarily affects elderly, and a concerning 22.9% viewed it as contagious (17).

### 4.3 Stroke risk factors identification

When asked about potential stroke risk factors, 25.2% of our participants were able to identify all risk factors, while 1.7% could not identify any. Around 91.6% recognized that hypertension is the primary stroke risk factor, followed by psychosocial stress (84.7%), high cholesterol (78.1%), old age (76.9%), and physical inactivity (73.1%). Our results can be interpreted by the fact that the American Heart Association’s 2021 guidelines list hypertension, hyperglycemia, obesity, renal dysfunction, and hyperlipidemia as key stroke risk factors, while also highlighting sedentary lifestyle factors such as smoking and poor diet as significant contributors (23). Recognition of risk factors often varied widely across studies, with 18 to 94% identifying at least one in open-ended questions and 42 to 97% in closed-ended formats (24). For instance, Dar et al. found that most respondents (26.8%) recognized two out of five risk factors, with 19.6% identifying all and 2.3% unable to identify any, consistently pointing to hypertension (93.5%) and diabetes mellitus (45.3%) as primary risk factors (17). Similarly, Sirisha et al. observed that most participants knew fewer than four risk factors, with about 8.85% unaware of any, noting psychological stress (57.6%) and hypertension (57.4%) as top risks (25). Yıldız et al. reported that only 6.5% knew all the risk factors, with hypertension (35.3%) and heart disease (18.5%) as main concerns (26). Lawrence et al. highlighted a concerning trend, with 53.3% of participants unaware of any risk factors, pointing to diabetes mellitus (28.6%) and hypertension (25.7%) as prevalent risks (27). Additionally, a community-based survey across Gulf Cooperation Council (GCC) countries (Qatar, Saudi Arabia, Kuwait, Bahrain, UAE, Oman) emphasized smoking as a significant risk factor (28), underlining the diverse yet overlapping risk factors recognized across different populations and studies.

### 4.4 Stroke symptoms identification

As for early stroke symptoms, 24.7% of our participants could recognize all early symptoms of stroke, although 4.5% could not identify any. The most common symptom reported was sudden difficulty in speaking/understanding speech (89.7%), likely due to its immediate and noticeable nature, clearly indicating a serious neurological event. Healthcare providers also often emphasize its importance during routine check-ups and educational sessions, reinforcing its significance. Other warning symptoms reported were sudden loss of consciousness/fainting (86.7%), sudden weakness/numbness (84.2%), sudden dizziness (83.6%), and severe headache (79.9%). Meanwhile, Deepthi et al. reported that about 28.9% of participants were able to identify only one stroke symptom, 3% could not identify any, and only 9% could identify all six symptoms listed, with difficulty in speaking (59.4%) and weakness on one side of face/body (54%) being the most common (29). Similarly, Madae’en et al. found that the majority of their participants (31.6%) recognized two stroke symptoms, while only 2.5% listed all symptoms, and 12.7% could not identify any. Sudden loss of speech (54.7%) followed by sudden weakness of one side of the body (49.1%) were the most recognized symptoms (30). Contrasting these findings, Dar et al. observed that none of their respondents identified all symptoms, with 26.2% unable to identify any, and the most frequently listed symptoms being sudden onset of weakness or numbness of limbs (66.9%) and sudden onset of fainting (37.2%) (17). Lawrence et al. also found only 5.7% identified all warning symptoms, while the majority (42.8%) failed to recognize any, with weakness of the arm/leg (53.3%) and difficulty in balancing (23.8%) being the most frequently reported warning symptoms (27). In GCC countries, Kamran et al. noted that weakness of limbs (23%) and speech problems (21.7%) were the most reported symptoms (28).

### 4.5 Stroke consequences identification

When inquired about stroke’s potential consequences, a substantial majority of our participants (37.5%) were able to enumerate all possible outcomes, though a small proportion (3.8%) could not identify any. Notably, 91.9% of those surveyed recognized that stroke could result in movement/functional problems, likely due to their immediate and visible impact on daily life. Additionally, 90.9% acknowledged the risk of long-term disabilities, 83.3% believed stroke could lead to cognitive/memory problems, and 77 and 70.5% anticipated visual problems and emotional or personality changes, respectively. Similarly, Madae’en et al. reported that the loss of ability to speak (62%) and walk (52.6%) were the most recognized consequences of stroke (30). In contrast, Alhazzani et al. found that death (63.2%) and paralysis (54.6%) were the most frequently reported outcomes of stroke by participants (31).

### 4.6 Predictors associated with the identification of stroke risk factors, early symptoms, consequences, and decision-making for hospital visits

In examining the predictors for identifying stroke risk factors, early warning symptoms, consequences, and decision-making for hospital visits, our study identified several key trends. For predictors related to identification of stroke risk factors, females were found to have a higher propensity than males to recognize risk factors, a finding supported by Reeves et al., who noted significantly greater awareness among women (32), probably due to their deeper interest in health issues, spending more time seeking health information than men (33). Employment also emerged as a significant predictor; employed individuals were more likely to identify stroke risk factors than their unemployed peers. This correlation could be due to the financial stability of employed persons, enabling better access to health information and more frequent healthcare consultations, as seen in a similar Spanish study (34).

In analyzing factors that influence the recognition of stroke’s early symptoms, females were significantly better than males at identifying at least one early symptom, confirming literature that associates female gender with greater awareness of stroke symptoms (35–37). In contrast, Wahab et al. found that males had superior knowledge of these symptoms (38). Consistent with the literature (35, 39–43), younger individuals were found to be more capable of recognizing stroke symptoms, likely due to better access to health information through digital platforms and social media. Interestingly, married individuals were less adept at recognizing symptoms, possibly due to distractions of familial obligations and lifestyle changes related to marriage, including diet and exercise habits, which could detract from health awareness. Furthermore, individuals with a university education were more capable of identifying symptoms compared to those with only a school education, a trend supported by other studies (20, 22, 35, 38, 44–47), probably due to higher health literacy. Individuals with a high income were significantly more likely to identify early stroke symptoms, possibly because higher income levels often correlate with better access to healthcare resources and information, allowing these individuals to receive regular medical check-ups and education. These results were also corroborated in studies by Yoon et al. (40) and Reeves et al. (32). People with chronic diseases like hypertension and obesity were more likely to identify early stroke symptoms, probably because they were more informed about health due to regular medical visits (48, 49) and actively seeking health information online (50). Hypertension was demonstrated to be a significant predictor of increased knowledge of stroke symptoms in research by Pancioli et al. (35), Schneider et al. (43), and Reeves et al. (32). Conversely, individuals with depression were found to be significantly less likely to identify early stroke symptoms compared to those without depression. Depression impairs cognitive function, reducing the ability to process and retain health information (51). It also lowers motivation to seek and engage with health education resources. Additionally, individuals with depression may interact less frequently with healthcare providers due to hopelessness or lack of energy, resulting in reduced exposure to health information (52).

In identifying stroke consequences, females were more capable than males, demonstrating a gender disparity in health awareness, despite inconsistent findings in previous gender-specific studies (53–55). Age was a crucial factor, with those aged 50 and above more likely to identify consequences than younger individuals, contradicting findings by Ramirez-Moreno et al. that older patients were less knowledgeable (34). Older adults are usually more aware of their higher risk for stroke and other health conditions and are more likely to have had personal or family experiences with stroke, making them more attentive to information about stroke consequences. Geographical differences also impacted awareness, with urban residents more likely to identify stroke consequences than rural ones. Alluqmani et al. found that urban residents were more likely to recognize consequences, likely due to better access to resources and health services in urban areas (56). Lifestyle choices and chronic conditions such as smoking and peptic ulcer disease positively enhanced awareness, likely because affected individuals were more attuned to their healthcare needs and the importance of understanding potential health risks, motivating them to educate themselves about conditions like stroke. Nonetheless, further research is needed to fully explore these aspects.

Regarding the decision to transport a patient experiencing stroke symptoms to hospital, our research identified several key determinants. Previously, limited research has focused on witness factors rather than patient factors affecting response behaviors to stroke, highlighting how environmental context and resources, social influences (e.g., prompts from patients), and beliefs about consequences shape witness behavior (57–59). Our study builds on this by identifying additional factors impacting behavior in response to stroke. Age was a significant factor; individuals aged 50 and above were less likely to transport patients to hospital compared to under 30, possibly due to generational differences in health education, leading older adults to underestimate stroke symptoms or delay seeking immediate medical intervention. Additionally, mobility and transportation challenges that worsen with age, along with potential skepticism toward the healthcare system, may also contribute to this reluctance. Geographical disparities also influenced decisions, with rural residents less likely to seek hospital care for a patient possibly due to limited healthcare access, transportation challenges, and lower stroke awareness. Marital status also impacted the decision-making process, with divorced and widowed individuals less likely to transport patients to hospital than singles, potentially due to the emotional and psychological impacts of their situations affecting their emergency response capabilities. Educational attainment seemed to positively influence decisions, with university educated individuals more inclined to take patients to the hospital compared to those with only school education. This trend was also noted by Dar et al., who observed that higher education was linked to a greater likelihood of taking a stroke patient to a hospital (17). Additionally, caregivers with chronic conditions like hypertension, peptic ulcer disease, and depression were less likely to transport patients, possibly due to physical or emotional barriers these conditions create, which could limit their physical ability to assist others in emergencies.

This study had several limitations. Firstly, the use of an anonymous online survey and the snowball sampling method may introduce selection bias, as it primarily reaches individuals with internet access and those active on social media platforms. This can skew the sample toward younger, more educated, and urban populations, potentially underrepresenting older adults, those with lower educational levels, and rural residents. Secondly, the cross-sectional design limits the ability to establish causality between observed factors and stroke awareness. Thirdly, self-reported data are subject to recall bias and social desirability bias, where participants might overreport their knowledge to present themselves in a better light. Lastly, the exclusion of individuals with a history of stroke may overlook insights from those who have firsthand experience with the condition, potentially missing valuable perspectives on stroke awareness and knowledge.

## 5 Conclusion

This study provides a comprehensive assessment of stroke knowledge and awareness among Arabic-speaking adults across seven countries in the MENA region. The findings reveal high levels of awareness regarding stroke risk factors and consequences, with notable variability in the identification of early symptoms across countries. Females, employed individuals, urban residents, and those with higher educational attainment demonstrated better knowledge of stroke-related information. The study underscores the importance of community-based educational initiatives to improve stroke awareness, early detection, and timely response. Enhancing public knowledge through mass media campaigns, educational programs, and healthcare initiatives is crucial for reducing the burden of stroke-related morbidity and mortality in the MENA region. Future research should address the identified limitations and explore strategies to reach underrepresented populations, ensuring a more inclusive approach to stroke education and prevention.

## Data Availability

The raw data supporting the conclusions of this article will be made available by the authors, without undue reservation.
